# Health Care Professionals' Knowledge of Stalking Perpetrators, Victims, Behaviors, and Coping Strategies: A Preliminary Study among Italian Hospitals

**DOI:** 10.1155/2019/9190431

**Published:** 2019-10-13

**Authors:** Daniela Acquadro Maran, Barbara Loera, Alberto D'Argenio

**Affiliations:** ^1^Work and Organizational Well-being Research Group, Department of Psychology, University of Torino, Via Verdi 10-10124 Torino, Turin, Italy; ^2^Department of Psychology, University of Torino, Via Verdi 10-10124, Turin, Italy; ^3^Policlinico Tor Vergata, Viale Oxford, 81-00133 Rome, Italy

## Abstract

The aim of this study was to investigate health care professionals' level of in-depth understanding about the various types and characteristics of stalking. In particular, the study examines knowledge on the characteristics of stalkers and their victims, acted behaviors, and coping strategies used to stop the harassment. The data were collected by means of an *ad hoc* questionnaire. The sample comprised 210 participants working in local health units in Turin, a large city located in the northern part of Italy. The majority were women (160, 76.2%). The participants were aged 20–64 years, and the mean age was 41.63 years (SD = 11.18). The majority of participants were psychologists (99, 47.1%), 31 (14.8%) were nurses, 31 (14.8%) had an unspecified medical profession, 29 (13.8%) were psychiatrists, and 20 (9.5%) were general practitioners. According to the findings, interventions with male victims of stalking, especially when the stalker is a woman, require attention in particular. Underestimating the stalking experience is a risk, so health care professionals in their interventions must explain to the men the emotive and physical consequences of the victimization. Moreover, in suggesting coping strategies, health care professionals must consider the victim's fear of reporting the incident not only to law enforcement authorities but also to family and friends. The findings showed that health care professionals need a better understanding of the stalking phenomenon. Education courses are a valuable tool to identify characteristics of the phenomenon, validate existing knowledge, and decrease the level of missing information to develop the skills needed to take appropriate action in cases of stalking.

## 1. Introduction

The term *stalking* is used to indicate a constellation of behaviors in which one individual persistently inflicts repeated, unwanted intrusions and communications on another person [1]. Stalking, which is characterized by intrusive acts (e.g., threatening, following, spying, receiving unwanted calls, and e-mails), creates a sense of loss of control in the victim, which might lead the victim to believe that he/she is living in an unsafe environment [2]. The consequences for the victim include physical, emotional, and social ramifications on well-being and quality of life [3, 4]. Victims live in a state of continuous threat even when the behavior is not marked by an explicit threat or by physical violence [5, 6].

One of the recommended strategies to reduce the impact of stalking on the life of the victim is to approach a health care professional (HCP) [7], with whom a relationship may facilitate the victim in clarifying, leading him/her to adopt more effective coping strategies, such as assertive communication when direct confrontation with the stalker is recommended. As reported by Kamphuis and his colleagues [8], the intervention of professionals in stalking cases is often based on their knowledge of the phenomenon. In some cases, the stalking campaign may be assessed as “merely a nuisance.” A risk is underestimating the stalking's effects on the victim, especially when the boundaries of the phenomenon do not fall within the typical stereotypes, such as the end of a romantic relationship or the genders of the stalker and victim [9]. Studies on the general population showed that the highest victimization prevalence type is that of the male perpetrator–female victim relationship [3, 10–12]. Moreover, when the stalker is a woman, underestimating the severity of the event, wherein the perpetrated actions and the consequences experienced by male victims are perceived to be less dangerous, is a greater risk. Cattaneo et al. [13] investigation demonstrated the difficulties of male and female victims experience in receiving help from social and health services. Their study, which involved 82 victims interviewed monthly over 7 months, showed that practitioners are often unsure how to address stalking. As underlined by Nikupeteri [14], “in working with stalking victims, professionals often lack knowledge about what constitutes stalking and thus often misunderstand or ignore its complex nature” (p. 794). Thus, knowledge on the impact of the stalker's misconduct on the victim's life is important in planning both an intervention that the victim may find helpful and one that will stop the stalkers.

Mullen and his colleagues [7] and Storey et al. [15] have suggested assessing and managing the stalking situation while considering the stalker's risk profile (e.g., the presence of a psychopathology), the behaviors acted (e.g., threatening), the type of motivation driving the stalking (e.g., reconciliation), the predisposing factor initiating a stalking behavior (e.g., need for attention), and the victim's risk profile, in particular the coping strategies adopted (e.g., purchasing a weapon). Regarding the presence of a psychopathology, Meloy at al. [16], who investigated a forensic population of 59 obsessional followers charged with the crime of stalking, found that their diagnoses included mood disorders, adjustment disorders, psychotic disorders, anxiety disorders, substance use disorders, and personality disorders, per the DSM criteria. In contrast, Galeazzi et al. [17] found that, in the general population, a primary DSM-IV diagnosis was reported only for 38 out of 361 stalkers of mental health professional. Nijdam-Jones et al. [18] studied a community-based sample with 137 stalking offenders and found that 72% percent met criteria for a clinical diagnosis. They also noted high rates of comorbidity: personality disorder (50%), substance use disorder (46%), mood or anxiety disorder (31%), and psychotic disorder (10%). Their findings showed the absence of a psychopathology for 28% of the sample. The findings of an early investigation conducted by Manunza and Pintor [19] in the Italian context showed that, from the forensic population, 20 stalkers (40%) had received a diagnosis of substance use disorder, 6 (30%) a diagnosis of bipolar and related disorders, and 5 (25%) a diagnosis of spectrum disorders of schizophrenia or other psychotic disorders. Moreover, 45% of stalkers were affected by not otherwise specified personality disorder. Clearly, the prevalence rates of psychopathology are dependent on the setting where it is measured (e.g., forensic versus general population), but more importantly, these findings in stalkers could indicate the correlation of a psychopathology with more persistent and recurrent stalking behaviors [20].

A stalker's misconduct could vary from a range of behaviors, from obsessive acts to behaviors that make the victim afraid or concerned for his or her safety [21], such as harassment and intimidation, surveillance tactics, and invasion tactics [22–25]. In regard to the type of motivation for stalking, Melton [26] suggested that stalkers may be motived by the inability to form a relationship, the desire to establish or reestablish a relationship, revenge in the context of a failed relationship, and the redress for a perceived wrong and/or sexual gratification. The predisposing factor initiating a stalking behavior was investigated by authors such as Lowenstein [27] and Thompson et al. [28], who argued that predisposing (e.g., sociocultural, psychological, and historical) and contextual (e.g., triggering events and disinhibition) factors influence the stalking behavior. Kienlen et al. [29], as well as Dennison and Stewart [30], stated that feelings such as anger and jealousy, a traumatic event in childhood, and the need for control predicted stalking behaviors. In regard to coping strategies, Amar and Alexy [31], in their descriptive study involving 262 college students, identified the most common coping strategies employed as the following: ignoring the problem; minimizing the problem; distancing, detaching, or depersonalizing; using verbal escape tactics; attempting to end the relationship; controlling the interaction; and restricting accessibility. Moreover, Davis et al. [32] noted the importance of emphasizing alcohol and drug use as negative (and counterproductive) methods of coping with stress in interventions with victims of stalking.

The aim of this study was to investigate HCPs' level of in-depth understanding about the various types and characteristics of stalking. In particular, the study examines knowledge regarding the characteristics of stalkers and their victims, acted behaviors, and coping strategies that are useful to stop the misconduct. Understanding what HCPs know of the phenomenon may help plan better-quality education for this kind of population. After taking into account their current knowledge of the phenomenon, focusing on the most useful aspects will help them better recognize the phenomenon and take action to help the victims, regardless whether the stalkers are men or women. To the best of our knowledge, this study is the first to investigate Italian HCPs' awareness of the stalking phenomenon.

## 2. Method

### 2.1. Ethical Statement

The study presented in this article followed all ethical guidelines required for conducting human research, including adherence to the legal requirements of Italy and compliance to the provisions of the 1995 Declaration of Helsinki, revised in Edinburgh, 2000 [33]. The research project was approved by the Board of Directors of the two Italian local health units (aziende sanitarie locali (ASL)) involved. Because the study does not provide medical treatment or procedures that will cause the participants psychological or social discomfort, additional ethical approval was not required. With the approval of the Board of Directors, department chiefs from each unit were asked for authorization to administer the questionnaire. Because the cover sheet clearly explained the research aim, the voluntary nature of participation, the anonymity of the data and the analysis of the findings, and filling in the questionnaire served as the participant's consent. Participants volunteered in the research without receiving any reward.

### 2.2. Measures

With the aim to explore the HCPs' knowledge about various aspects of stalking, the data were collected by means of an *ad hoc* questionnaire. In section two for the perpetrated behavior, a modified Italian version of the questionnaire constructed by the Network for Surviving Stalking with forensic psychologist Dr. Lorraine Sheridan, University of Leicester, was used. The Italian version of the questionnaire has already been used in a previous investigation among nurses [34] and HCPs [35]. Every participant received a self-administered anonymous questionnaire. A short description of the research study was provided on the first page to explain the aim of the research and provide instructions on how to fill out the questionnaire.

The questionnaire is divided into 3 sections:

- Section 1 asked the participants for their demographic data (e.g., gender, place of residence, age, marital status, and profession).
- Section 2 focused on the professionals' knowledge of stalking and contained questions on the gender prevalence of the stalker population (i.e., the probability that a stalker is a female and/or a male; from 0–10, 11–20, 21–30, 31–40, 41–50, 51–60, 61–70, 71–80, 81–90, to 91–100); the probability of a psychopathology related to stalking behavior based on the DSM-IV in the context of a specific clinical diagnosis (mood disorder, adjustment disorder, psychotic disorder, anxiety disorder, substance use disorder, personality disorder, and no diagnosis); perpetrated behaviors (18 items, e.g., “waiting outside home”; 1 = totally disagree and 5 = totally agree); and the motivations that determined the initiation of the stalking campaign (8 items, e.g., “revenge”; 1 = totally disagree and 5 = totally agree). Moreover, participants were required to indicate the predisposing factor initiating a stalking campaign (14 items, e.g., “childhood trauma”; 1 = totally disagree and 5 = totally agree).
- Section 3 was dedicated to the stalkers' victims. In this case, respondents were asked to identify the percentage of prevalence of male victims and female victims within specific ranges (i.e., the probability that a victim is a female and/or a male; from 0–10, 11–20, 21–30, 31–40, 41–50, 51–60, 61–70, 71–80, 81–90, to 91–100). They were also requested to indicate the strategies used to cope with harassment (9 items, e.g., “report to police”; 1 = totally disagree and 5 = totally agree).

### 2.3. Procedure

Participants were recruited at a seminar on “stalking and helping professions” held by one of the authors (University of Turin) at two local health units operating in Turin, Italy, during the months of October and December 2016. The seminar was aimed at medical professionals, nurses, psychologists, and psychiatrists and presented the stalking phenomenon and strategies to handle it. The seminar was presented in two sessions, one for each local health unit. The questionnaire was administered before the beginning of the seminar and was filled in by all of the participants. The session in October 2016 had 110 participants, and the second session, held in December 2016, had 100.

### 2.4. Statistical Analysis

The statistical analysis was carried out using the Statistical Package for the Social Sciences (SPSS v. 25). Data were subject to preliminary descriptive analyses. Appropriate inferential statistic tests (chi-squared, *t*-test) and one-way ANOVA models (*F*-test) were used to identify associations between responses and the scale levels of the examined variables. Finally, to understand the structure of the professionals' belief systems in relation to *predisposing factors* and *characterizing behaviors*, the direct Oblimin method with oblique rotation and Kaiser normalization were used to analyze the main components.

## 3. Results

The sample comprised 210 participants working in local health units in Turin, a large city located in the northern part of Italy. The majority were women (160, 76.2%); the data confirmed the gender trend in health professions [36]. The participants were aged 20–64 years, and the mean age was 41.63 years (SD = 11.18). The majority of participants were psychologists (99, 47.1%), 31 (14.8%) were nurses, 31 (14.8%) had an unspecified medical profession (referred to as “others”), 29 (13.8%) were psychiatrists, and 20 (9.5%) were general practitioners.

### 3.1. HCPs' Knowledge of Stalking Characteristics

First, participants were asked to attribute a gender to the stalker while also expressing the likelihood of the stalker being a man rather than a woman. Two 0–100 scales were used to facilitate the cognitive tasks expressing the percentage of each gender in the total number of stalking crimes. Respondents reported the gender prevalence of the supposed stalker in overall crimes. The majority of respondents stated that the stalker is a man in almost 70% of the cases. By using the median as the spread cutoff (female stalkers: 21–30% of cases; male stalkers: 71–80% of cases), the two judgments on stalker gender prevalence were dichotomized and combined by type (Table 1).

Only 33 HCPs (15.7%) indicated the stalker as a female, whereas the majority of respondents (40%) reported the stalker as belonging to the male gender. The remaining respondents were divided between those who refused to indicate any gender prevalence because they believed the male or female gender to be equally possible (21%) and those who simply did not indicate any gender prevalence at all (23.3%). There was no association between the gender of the respondent and the gender of the stalker (male: *T* = 0.219, *p*=0.827; female: *T* = 0.645, *p*=0.520).

**Figure 1:**
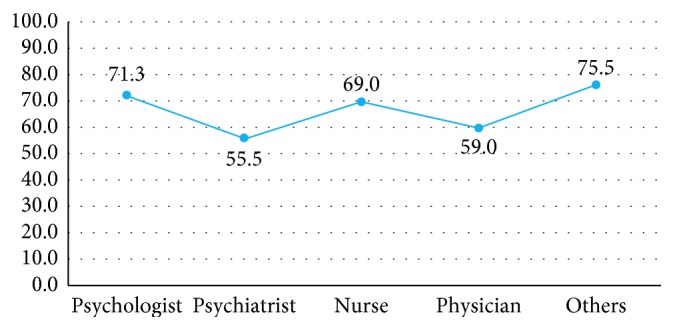
Prevalence of male stalkers by professional role.

The type of profession is not associated with the female gender prevalence but does seem to be attributed to assuming a stalker is male (*F* = 4.103, *p* < 0.05); psychologists, nurses, and other professionals in particular believe the stalker to be a man typically (Figure 1).

The results indicated a strong relationship between the gender attributed to the stalker and the one attributed to the victim (*χ*^*2*^ = 246.85, *Cramer's V* = .63, *p* < 0.001). The interviewed professionals thought that stalking was primarily committed by men against women (81%) and, on a smaller scale (61.5%), by women against men (Table 2).

When asked to attribute characterizing psychopathological disorders to stalkers, respondents said that stalkers may have personality disorders (80%) and adjustment disorders (43%). The other suggested diseases (mood disorders, psychosis, anxiety, and addiction) were equally attributed in percentages equal to or lower than 25% (Table 3). No differences in attribution characterization related to the stalker's gender were observed. With the exception of mood disorders, which were mainly attributed by HCPs who did not indicate a gender prevalence for the stalkers (*F* = 2.91, *p* < 0.05), there was no significant association between the stalker's gender and the diagnosis suggested by the respondents.

### 3.2. Stalkers' Risk Factors and Behavioral Typicality: HCPs' Beliefs

**Figure 2:**
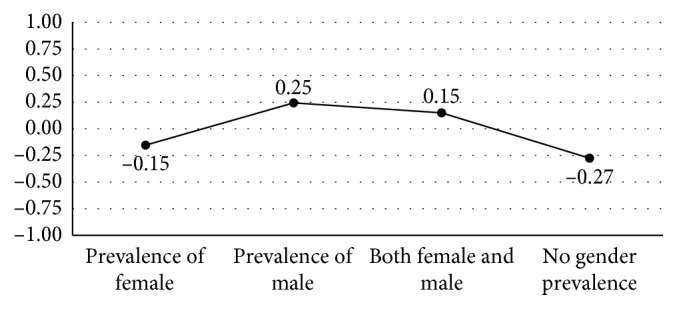
Health care professionals' beliefs about surveillance tactics typicality in relation to the gender prevalence of the stalkers (mean score).

Data related to typical stalker behaviors were analyzed using exploratory factor analysis (extraction method: main components; rotation: Oblimin rotation with Kaiser normalization; Table 4). The first component summarized the participants' responses to items attributed to aggression-related behaviors and to aggression threats toward the victim or third parties (e.g., family members, partner, and children). It also included items associated with the act of visiting the victim's home, which, according to the literature, is typical of the ex-intimate stalker [24, 37]. This first component is labeled *harassment and intimidation*. This label is in accordance with Cupach and Spitzberg [24], who noted that harassment and intimidation represent a variety of aggressive verbal or nonverbal activities designed to bother, annoy, or otherwise stress the victim. The second component, named *surveillance tactics*, summarizes five items related to surveillance activities aimed at collecting information and controlling the victim. In this case, the semantic structure is also in line with studies conducted by Yanowitz and Yanowitz [38], who identified a particular form of the stalking phenomenon in these behaviors. The third factor includes behaviors that, according to Spitzberg's classification [39], are aimed at privacy violation and invasion of the victim's personal space (*invasion tactics*). Overall, the three factors accounted for 53.5% of the total observed variance of the responses. The ANOVA showed that the behaviors labeled *surveillance tactics* were reported by those who believed that stalking was mainly perpetrated by a male stalker (Figure 2); the same behaviors were not typical for those who did not identify a gender prevalence (*F* = 2.74, *p* < 0.05).

An analysis of the participants' responses concerning motivational factors resulted in a unidimensional solution (Table 5). The semantics of the factors revealed that HCPs first attribute the stalkers' campaign to egoistic motives connected to the redress and revenge of presumed wrongdoings to which they were subjected. Consequently, the items describing actions interpreted as constructive had negative factor loadings. No significant differences emerged between respondents who attributed a specific gender prevalence and the work they carried out.

In regard to predisposing factors, the results of the analysis on the main components are shown in Table 6. Responses to the items are organized in a three-category structure accounting for 51.2% of the total observed variance.

The first extracted component includes predisposing factors describing overexpression and dysregulation of affectivity—that is, the inability to manage the intensity and duration of negative emotions such as fear, sadness, or anger [40]. This first factor was labeled *affectivity dysregulation*. The second factor includes items related to cognitive functioning impairment. Nicastro et al. [41] emphasized that cognitive problems also include mental states such as confusion, distrust, suspiciousness, self-esteem issues, and suicide ideation. This factor was named *cognitive problem*. The third factor comprises social difficulties that could cause problems in relationships with others, including friends, family, and colleagues [42]. The third factor was named *social problem*. This factor is mainly indicated by the HCPs who consider stalking to be perpetrated by both genders.

### 3.3. Coping Strategies Adopted by the Victims

HCPs indicated that the most practiced coping strategy adopted by victims is rational changes in habits, which seem useful in reducing the risk of having contact with the stalker but also results in social inclusion (Table 7). In addition to these defensive choices, the HCPs indicated that victims can perform more assertive strategies, such as catching the stalker in the act, collecting evidence, and obtaining legal assistance. Maladaptive coping strategies are less cited. Professional roles determine the focus on certain strategies: psychologists and physicians, in contrast to nurses and psychiatrists (*F* = 3.0, *p* < 0.05), affirming that victims are used to adopting defensive strategies and escape from stalking campaigns by means of social isolation. Conversely, nurses and psychiatrists believed that victims often turn to lawyers and psychologists to obtain support (legal support: *F* = 2.74, *p* < 0.05; psychological support: *F* = 5.80, *p* < 0.01).

## 4. Discussion

The aim of this work was to describe the HCPs' knowledge about various aspects of stalking, the perpetrators, the victims, both of their behaviors, and coping strategies. Given the nature of this work, these professionals are informed of the victim's discomfort and are called upon to provide clinical treatment to the stalker. The research results may help in better defining information that can be useful for designing *ad hoc* training courses for this type of population and for providing information that will be useful in interventions to help the victim and to stop the stalker. The initial data focused on the gender prevalence of both the stalkers and victims. The data indicated by the HCPs are in line with what has emerged from previous studies on stalking, both about the genders of the victims [3, 12, 43] and the stalkers [10–12]. The findings showed that interventions with male victims of stalking, especially when the stalker is a woman [44], require attention in particular, as these cases run the risk of underestimating the stalking experience. HCPs in their interventions must explain the emotive and physical consequences of the victimization to their male victims. Moreover, in suggesting coping strategies, HCPs must take account of the fear victims may experience in reporting the stalking not only to law enforcement authorities but also to family and friends [45].

In regard to psychopathological disorders attributed to stalkers, the “no diagnosis” response, which was the option least indicated by the respondents, was an interesting finding. Galeazzi and Curci [46] highlighted only a 10% prevalence of a DSM-diagnosed disorder in stalkers. In our sample, the HCPs who did not indicate a certain prevalence in regard to the stalker's gender, on average, more frequently indicated a mood disorder. This is a result that should be brought to the HCPs' attention; Meloy and Gothard [47] suggested that a mood disorder presents in one out of every four stalkers. Findings from this investigation are in line with what was reported by Nijdam-Jones and his colleagues [18], who stated that the stalkers' psychopathology types varied. Another interesting finding is related to the motivation that initiates the stalking behavior. In this case, none of the results indicated any specific motivation for choosing a main reason for initiating the stalking. More information on the presence and absence of psychopathological disorders and on stalker motivations can help HCPs enhance their knowledge of the stalking phenomenon and find ways to intervene with victims and stalkers. For example, stalkers with mood disorders may be receptive to pharmacological and psychotherapeutic interventions such as therapy based on a functional analysis approach [18] or a cognitive approach [48]. At the same time, victims dealing with stalkers with personality disorders may find it harder to stop the stalking behavior, as such stalkers with such disorders tend to be resistant to treatment [47, 49]. Thus, HCPs conducting mental assessments need to be able to recognize which individual stalkers are more likely to persist in harassing their victims.

The typical behavior results reflected the characteristics of the stalking phenomenon. Generally, stalking behaviors include harassment and intimidation, surveillance tactics, and invasion tactics, as suggested by Sheridan et al. [22], Spitzberg and Cupach [23, 24], and Brady and Nobles [25]. We suggest that HCPs pay attention to behaviors that may be considered a warning sign of a possible escalation from intimidation to a physical threat—that is, when aggressiveness is not only expressed as potential violence but also shows the characteristics of perpetrated violence. This is particularly useful for those HCPs who indicate stalking to be mainly perpetrated by a man and who, on average, mainly indicate surveillance tactics as a stalking behavior.

As regards the predisposing factor, affectivity dysregulation is, as previously described, the manifestation of a maladaptive underregulation of emotions. Stalking behavior is linked to the mismanagement of one's own emotions, which manifest in the attack against the person who is the cause of the perceived discomfort [40] or the victim. Cognitive problems can lead to imprecise thoughts and thought processes that support maladaptive behaviors, minimizing one's responsibility for the act [50]. Stalkers, for example, believe that their behaviors do not harm the victim, that society rules can be ignored, and that women or men are deceptive beings. Regarding the social problems factor, the explanation of stalking can be found in impaired or conflicting social relations [51]. Because this factor is mainly indicated by HCPs who consider stalking to be a phenomenon perpetrated by both genders, it is important to highlight all aspects of the phenomenon in a training course. Otherwise, one may risk excessively focusing on one aspect to the detriment of varied factors that can lead to the initiation of stalking behaviors.

One final finding concerns the coping strategies that HCPs indicated are used by stalking victims. Interestingly, nurses who more frequently identify strategies such as seeking legal advice, requesting psychological support, and increasing alcohol consumption are more inclined to consider the victim as a person who can use different strategies to escape the victimization condition. Most likely, these data are ascribable to the nursing profession. Direct personal knowledge of the stalking phenomenon may be an explanation. As shown by previous research studies [52–54], nursing professionals are particularly at risk of stalking. The nurses may have been stalking victims themselves, and consequently, the experience described may resemble their own personal experiences. Because participants spontaneously participated in the training course, there may be a selection bias. As it is a sensitive issue, participation may be influenced by the individuals' sensitivity toward the topic or by them viewing participation as an opportunity to rethink their victimization experience. Future research should include a questionnaire with a section devoted to the stalking victimization experience or other victimization phenomena (e.g., sexual harassment and domestic violence). In fact, if a previous victimization experience has not been processed, it may bias the knowledge of the phenomenon and of the parties involved. As Guldimann and his colleagues [55] showed, the contradiction of being both victim and care provider in victimization cases may result in minimizing or denying the problem.

The research presented here has some limitations. First, the survey was distributed before two seminars on the topic. The risk of bias is high since it is likely that attendees had a special interest in the topic. To better understand HCPs' knowledge about the phenomenon, future research should consider comparing HCPs who attended the seminar to those who did not. Second, the sample does not represent the entire HCP population. Therefore, the results cannot be generalized. As previously described, a selection bias may partly describe the results, especially in regard to the professional category of nurses. In this respect, it may be useful in the future to provide information beforehand to better clarify the purpose of the questionnaire and of the professional's participation in the training course. Another limitation is that some important variables related to stalking have not been considered. For example, we have not inquired about the victims' sexual orientation. Therefore, we have not investigated how the phenomenon is perceived in the lesbian, gay, bisexual, and transgender (LGBT) communities. Future research should also attempt to identify, in addition to the prevalence of the victim's and the stalker's genders, the nature of the perpetrator-victim relationship. This information could be useful for better understanding the phenomenon, not only in terms of emotional relationships but also in friendships, simple acquaintanceships, or relationships between strangers. As noted by Quinn-Evans et al. [56], stalking is a phenomenon that could imply different motivations, not only affective ones.

## 5. Conclusion

Despite the limitations of the study, HCPs, without doubt, need to have more knowledge of the various aspects of the stalking phenomenon. This is fundamental also in view of the fact that, as suggested by Galeazzi and De Fazio [57], this population is itself at risk of victimization, in particular by patients. In their review of the literature, they found that “there is a high rate of professional victimization—more than 10% across different roles in mental health” (p. 57). Education courses are a valuable tool to identify the characteristics of the phenomenon, validate existing knowledge, and fill in missing information to increase the chances of providing appropriate assistance in cases of stalking [58, 59]. These courses must be tailored to this particular group of professionals and give information about the efficacy of intervention in victims (e.g., support groups and cognitive processing therapy) [60] and stalkers, regardless whether they have a psychopathology diagnosis or no diagnosis. For example, an intervention such as dialectical behavior therapy could be useful in particular for stalkers with a personality disorder; another useful intervention to consider is the Problem Behavior Program, which provides treatment to individuals with similar problem behavior, with or without the presence of mental illness [48, 60, 61]. Our research suggests that the various aspects of stalking should be presented to highlight the possibility that the perpetrator and the victim may be of the same gender and that the relationship between them may not fit into the female victim-male perpetrator relationship. Moreover, training courses should provide data on stalking behaviors, motivations underlying stalking behavior, predisposing factors, as well as the psychopathological characteristics of a stalker. We suggest designing a questionnaire that collects data on the HCPs' knowledge of the phenomenon. Similar to the study presented here, the questionnaire can be used as a tool to collect information about the participants' knowledge of the phenomenon to design a training program that fills in information gaps and validates existing knowledge.

## Figures and Tables

**Table 1:** Gender prevalence of stalkers.

			Male		Total
			No	Yes	
Female	No	Count	49	84	133
		%	23.3	40.0	63.3
	Yes	Count	33	44	77
		%	15.7	21.0	36.7
Total		Count	82	128	210
		%	39.0	61.0	100.0

**Table 2:** Stalkers' and victims' genders as indicated by health care professionals (*N* = 210).

Stalkers	Victims							
	Prevalence of female		Prevalence of male		Both female and male		No gender prevalence	
	*N*	%	*n*	%	*n*	%	*n*	%
Prevalence of female	8	12.3	40	61.5	14	2.5	3	4.6
Prevalence of male	34	81.0	2	4.8	5	11.9	1	2.4
Both female and male	13	20.3	4	6.3	47	73.4	0	0.0
No gender prevalence	7	17.9	4	10.3	2	5.1	26	66.7

**Table 3:** The stalker's psychopathology diagnosis according to the health care professionals (*N* = 210).

	*M*	SD
Mood disorder	2.65	1.16
Adjustment disorder	3.10	1.26
Psychotic disorder	2.61	1.28
Anxiety disorder	2.64	1.21
Use of substance disorder	2.50	1.17
Personality disorder	4.23	1.03
No diagnosis	2.18	1.41

*Note*. *M* = mean; SD = standard deviation.

**Table 4:** Factorial analysis of the stalkers' behavioral typicality: the structure of health care professionals' beliefs.

	*F1*	*F2*	*F3*
	*Harassment and intimidation*	*Surveillance tactics*	*Invasion tactics*
1. Sexual aggression	0.910		
2. Physical assault	0.869		
3. Threat of sexual aggression	0.757		
4. Threat of harassment of third parties	0.750		
5. Threat of physical assault	0.744		
6. Harassment of third parties	0.659		
7. Home visiting	0.638		
8. Waiting outside home		0.898	
9. Following		0.872	
10. Visiting workplace/school		0.798	
11. Unwanted communication		0.700	
12. Spying		0.638	
13. Deceiving			0.843
14. Sending unwanted gift			0.719
15. Manipulating			0.699
16. Spreading lies			0.682
17. Property damage			0.669
18. Communicating through a website		0.397	0.446

**Table 5:** Factorial analysis of the motives behind the stalking behaviors: the structure of health care professionals' beliefs.

	*F1*
Redress	0.926
Reconciliation	−0.791
Revenge	0.723
Sexual gratification	0.711
Other motives	0.656
Start a relationship	−0.648
End of the relationship	0.500
Inability to form a relationship	0.491

*Note*. Principal component extraction.

**Table 6:** Factorial analysis of the predisposing factors for the stalking behaviors: the structure of health care professionals' beliefs.

	Affectivity dysregulation	Cognitive problems	Social problems
1. Atypical view of love	0.849		
2. Anger	0.761		
3. Violent attitude	0.685		
4. Frustration	0.654		
5. Jealousy	0.386		
6. Insecurity	0.373	0.334	
7. Fear of abandonment		0.725	
8. Need for attention		0.698	
9. Low self-esteem		0.649	
10. Desire for control		0.624	−0.305
11. Low cultural level			0.844
12. Abuse of alcohol and drugs			0.659
13. Social maladjustment	0.387		0.570
14. Childhood trauma		0.324	0.375

*Note*. Principal component extraction, direct Oblimin rotation, and Kaiser normalization; factors loading <0.3 omitted.

**Table 7:** Victim's coping strategies according to health care professionals.

	%
Change phone number	90.8
Obtain legal consult and assistance	69.2
Limit social relationships	65.2
Renounce social activities	57.8
Catch the stalker in the act and collect evidence	53.6
Request pharmacological support	51.4
Change home, job, and car	48.8
Require psychological support	45.9
Reinforce security by adopting precautionary behavior	37.3
Change identity and/or city	27.1
Drink alcohol	21.2
Buy a weapon	15.3
Consume drugs	7.7

*Note*. Frequency of citation.

## Data Availability

The data used to support the findings of this study are available from the corresponding author upon request.
